# An optimal strategy for epilepsy surgery: Disruption of the rich-club?

**DOI:** 10.1371/journal.pcbi.1005637

**Published:** 2017-08-17

**Authors:** Marinho A. Lopes, Mark P. Richardson, Eugenio Abela, Christian Rummel, Kaspar Schindler, Marc Goodfellow, John R. Terry

**Affiliations:** 1 Living Systems Institute, University of Exeter, Exeter, United Kingdom; 2 Wellcome Trust Centre for Biomedical Modelling and Analysis, University of Exeter, Exeter, United Kingdom; 3 EPSRC Centre for Predictive Modelling in Healthcare, University of Exeter, Exeter, United Kingdom; 4 Institute of Psychiatry, Psychology and Neuroscience, Kings College London, London, United Kingdom; 5 Support Center for Advanced Neuroimaging (SCAN), University of Bern, Bern, Switzerland; 6 Department of Neurology, Inselspital, Bern, Switzerland; Brain and Spine Institute (ICM), FRANCE

## Abstract

Surgery is a therapeutic option for people with epilepsy whose seizures are not controlled by anti-epilepsy drugs. In pre-surgical planning, an array of data modalities, often including intra-cranial EEG, is used in an attempt to map regions of the brain thought to be crucial for the generation of seizures. These regions are then resected with the hope that the individual is rendered seizure free as a consequence. However, post-operative seizure freedom is currently sub-optimal, suggesting that the pre-surgical assessment may be improved by taking advantage of a mechanistic understanding of seizure generation in large brain networks. Herein we use mathematical models to uncover the relative contribution of regions of the brain to seizure generation and consequently which brain regions should be considered for resection. A critical advantage of this modeling approach is that the effect of different surgical strategies can be predicted and quantitatively compared in advance of surgery. Herein we seek to understand seizure generation in networks with different topologies and study how the removal of different nodes in these networks reduces the occurrence of seizures. Since this a computationally demanding problem, a first step for this aim is to facilitate tractability of this approach for large networks. To do this, we demonstrate that predictions arising from a neural mass model are preserved in a lower dimensional, canonical model that is quicker to simulate. We then use this simpler model to study the emergence of seizures in artificial networks with different topologies, and calculate which nodes should be removed to render the network seizure free. We find that for scale-free and rich-club networks there exist specific nodes that are critical for seizure generation and should therefore be removed, whereas for small-world networks the strategy should instead focus on removing sufficient brain tissue. We demonstrate the validity of our approach by analysing intra-cranial EEG recordings from a database comprising 16 patients who have undergone epilepsy surgery, revealing rich-club structures within the obtained functional networks. We show that the postsurgical outcome for these patients was better when a greater proportion of the rich club was removed, in agreement with our theoretical predictions.

## Author summary

Epilepsy is a chronic neurological disorder that affects about 1% of people worldwide. The administration of antiepileptic drugs is the preferable treatment, but in around one third of cases, drugs do not stop seizures, and these patients are potential candidates for surgery. Epilepsy surgery however is too often unsuccessful, with around one half of patients continuing to experience seizures. In this work we use mathematical models to study epilepsy surgery so to inform surgeons concerning the brain tissue that should be considered for surgery resection. We show that functional networks derived from data of epileptic patients considered for surgery present rich-club organization. For this kind of network structure, we propose an optimal surgery strategy that consists of disrupting the rich-club.

## Introduction

Epilepsy is a chronic neurological disorder that affects about 1% of people worldwide [1]. Antiepileptic drugs are the preferred treatment, but in around one third of cases, drugs do not stop seizures, and patients for whom this is the case are potential candidates for surgery [2]. Surgeons use an array of data modalities, including intra-cranial electroencephalogram (iEEG), in an attempt to map regions of the brain thought to be crucial for the generation of seizures [3]. If these regions of the brain are amenable to surgery (e.g. they do not overlie eloquent cortex), then they are removed with the hope that the individual is rendered seizure free as a consequence. However, long-term success rates from surgery may be as low as 15%, presumably in part due to failures of the assumptions used in the decision making process [4,5]. It is therefore crucial to advance our understanding of the mechanisms that generate seizures and the reasons why removing regions of brain tissue may or may not lead to seizure freedom.

In this regard, seizures are increasingly recognised as arising in large-scale brain networks [6–9]. Emerging from such networks, both healthy and pathological dynamics are observed, for example through EEG, MEG or fMRI. These dynamics emerge due to the interplay between intrinsic properties of brain areas, structural connectivity, and modulating influences across multiple temporal and spatial scales [10–12]. This networks paradigm has led to imaging or electrographic data being used to inform network representations of the brain (for example structural or functional brain networks), and graph theoretical measures are used to characterise the topology of these networks [13–19]. Studies analysing graph theoretical properties of networks have reported differences between functional and structural networks derived from healthy individuals versus people with epilepsy [20–26].

The emerging field of dynamics on networks is complementary to these traditional, “static” network analyses [27,28], and moves beyond the study of the topology of networks. In this approach, mathematical models are used to link networks and the intrinsic properties of individual nodes to dynamic data [29], which provides an avenue to understand the relationship between structure and function [30]. In particular, mathematical models that can recreate elements of pathological dynamics, for example the occurrence of seizures, have been used to understand the network mechanisms of disorders such as epilepsy [9,31–37]. Such approaches are also being used in translational applications, for example providing additional information to complement clinical interpretation, namely within the diagnosis of epilepsy [35,38]. Crucially, a dynamics on networks approach can be extended to study perturbations to networks. On one hand, lesions and traumatic brain injury can lead to the emergence of pathological brain activity, on the other hand, perturbations such as pharmacological treatment, single pulse electrical stimulation (and other electrical stimulations), transcranial magnetic stimulation, thermocoagulation, among others, can transform brain dynamics from pathological to healthy states [36,39–41], therefore revealing potential avenues for therapy. In the case of epilepsy surgery, we have demonstrated that a network model derived from iEEG data could provide relevant predictions for the outcome of epilepsy surgery [42]. Our findings have been recently replicated in an independent cohort of 16 people with pharmacoresistant epilepsy [43] offering further support to a dynamics on network approach. However, the ways in which networks with different topologies respond to perturbations is at present unknown. For example, in analogy to epilepsy surgery, it is unclear whether particular networks are amenable to a reduction in pathological dynamics upon removing nodes and if so which nodes would be best to target.

Here, we use a dynamics on networks approach to study the generation of pathological activity in networks and how the removal of nodes can restore healthy dynamics. Our starting point is a neural mass model that has previously been shown to generate epileptiform rhythms in focal seizures [32,37,44], and that we have successfully used to quantify and predict the outcome of epilepsy surgery [42]. It has been shown that the model, when placed close to a saddle-node on invariant circle (SNIC) bifurcation, can generate spontaneous, recurrent transitions to epileptiform dynamics (both inter-ictal spikes as well as seizures) when driven by noise [32,37,42]. In our framework, the neural mass model describes the dynamics of a single node within a wider network. The systematic exploration of node removal in brain networks is computationally demanding, and hence we seek a computationally efficient version of this model that preserves the quantification of the effect of removing nodes. We show that a modification of the theta-neuron model [45] is appropriate for this purpose since it is the canonical form of the bifurcation under consideration. This model is capable of generating spiking dynamics, which here represents seizure-like activity.

The computational benefits of the theta-neuron model allow us to study the emergence of spiking dynamics in different types of networks and also to systematically quantify the effect of removing different nodes. Here, we study small-world, random, rich-club and scale-free and find that rich-club and scale-free networks more readily generate spiking dynamics, since they require a lower strength of coupling between connected nodes to do so. In terms of the contribution of nodes, we find that rich-club, random and scale-free networks possess a small number of nodes that drive spiking dynamics, whereas the propensity of generate spiking dynamics is more evenly distributed across nodes in small-world networks. Collectively, this suggests that patients whose brain networks display rich-club properties should be particularly amenable to current surgery paradigms. In order to test the relevance of these findings, we analyse data from patients who underwent surgery and for whom postoperative outcome is known. We demonstrate that functional networks inferred from iEEG during seizures display a rich-club connectivity structure and that the proportion of rich-club nodes removed correlates with the success of surgery.

## Methods

### Ethics statement

This study was approved by the Internal Review Board of the Inselspital (approval No. 159399, dated 26th of November, 2013). All patients gave written informed consent that imaging and EEG data may be used for research purposes.

### Wendling model

In order to model epilepsy surgery, we consider large-scale brain networks, where each network node is capable of generating epileptiform activity but will do so depending on the connectivity structure of the network. In this framework, a node putatively represents a portion of brain tissue potentially responsible for the emergence of seizure activity across the network. We assume that the dynamics of each node can be described by a neural mass model, such as the Wendling model [37,42]. The model depicts the dynamics of a macroscopic circuit in which a population of excitatory pyramidal neurons interacts with three populations of interneurons (representing one excitatory and two inhibitory populations). The two inhibitory populations are classed as slow and fast, representing dendritic-projecting GABA_A_ and somatic-projecting GABA_A_ interneurons, respectively. The dynamics is described by the following 10 first-order ordinary differential equations (ODEs):

$${\dot{z}}_{1}(t)=z_{6},$$

$${\dot{z}}_{2}(t)=z_{7},$$

$${\dot{z}}_{3}(t)=z_{8},$$

$${\dot{z}}_{4}(t)=z_{9},$$

$${\dot{z}}_{5}(t)=z_{10},$$

$${\dot{z}}_{6}(t)=AaS\{z_{2}(t)-z_{3}(t)-z_{4}(t)\}-2az_{6}(t)-a^{2}z_{1}(t),$$

$${\dot{z}}_{7}(t)=Aa(p+C_{2}S\{C_{1}\;z_{1}(t)\})-2az_{7}(t)-a^{2}z_{2}\;(t),$$

$${\dot{z}}_{8}(t)=BbC_{4}S\{C_{3}z_{1}(t)\}-2bz_{8}(t)-b^{2}z_{3}(t),$$

$${\dot{z}}_{9}(t)=GgC_{7}S\{C_{5}\;z_{1}(t)-z_{5}(t)\}-2gz_{9}(t)-g^{2}z_{4}(t),$$

$${\dot{z}}_{10}(t)=BbC_{6}S\{C_{3}z_{1}(t)\}-2bz_{10}(t)-b^{2}z_{5}(t),$$

where *z*_1_-*z*_5_ are the output potentials in mV of the neuronal populations, namely *z*_1_, *z*_2_, *z*_3_, and *z*_4_ are the outputs of the pyramidal cells, excitatory population, slow inhibitory population, and fast inhibitory population, respectively. *z*_5_ is the output of the slow inhibitory population that interacts with the fast inhibitory population. *z*_6_-*z*_10_ are auxiliary variables, *S* is a sigmoid function,

$$S(\nu )=\frac{2e_{0}}{1+e^{r(\nu_{0}-\nu )}},$$

and *A*, *a*, *B*, *b*, *G*, *g*, *C*_1_-*C*_7_, *p*, *e*_0_, *r*, and *ν*_0_ are parameters (see Table 1 for their biophysical interpretation and values).

**Table 1:** Model parameter values and biophysical interpretations.

Parameter	Interpretation	Value
*A*	Mean excitatory synaptic gain	5 mV
*B*	Mean slow inhibitory synaptic gain	40 mV
*G*	Mean fast inhibitory synaptic gain	20 mV
*a*	Inverse average time constant–excitatory feedback loop	100 /s
*b*	Inverse average time constant–slow inhibitory feedback loop	50 /s
*g*	Inverse average time constant–fast inhibitory feedback loop	500 /s
*C*_1_	Connectivity strength–pyramidal to excitatory	135
*C*_2_	Connectivity strength–excitatory to pyramidal	0.8 *C*_1_
*C*_3_	Connectivity strength–pyramidal to slow inhibitory	0.25 *C*_1_
*C*_4_	Connectivity strength–slow inhibitory to pyramidal	0.25 *C*_1_
*C*_5_	Connectivity strength–pyramidal to fast inhibitory	0.3 *C*_1_
*C*_6_	Connectivity strength–slow inhibitory to fast inhibitory	0.1 *C*_1_
*C*_7_	Connectivity strength–fast inhibitory to pyramidal	0.25 *C*_1_
*ν*_0_	Firing threshold potential	6 mV
*e*_0_	Half of maximum firing rate of neural masses	2.5 /s
*r*	Slope of potential to rate sigmoid at *ν* = *ν*_0_	0.56 /mV

The values were established in [46], excluding *A*, *B* and *C*.

The output of the model *z*_2_(*t*)–*z*_3_(*t*)–*z*_4_(*t*) corresponds to the aggregated membrane potential of the excitatory cell population and its bifurcations have been extensively characterized [47]. In particular, a SNIC bifurcation has been identified as one mechanism for the generation of epileptiform rhythms observed in typical focal epilepsies [32]. This model and bifurcation were also previously employed to estimate brain network ictogenicity to predict the outcome of epilepsy surgery [42]. Therefore the parameters *A*, *B* and *C* were chosen so that the neural mass is in a steady state close to the SNIC bifurcation that gives rise to spiking dynamics which we consider a proxy for the patho-phenotype of the epileptic brain (see the third figure, left panel, in [32]). *p* is an extrinsic input parameter that represents stimuli from other areas of the cortex.

Although the neural mass model described above represents the dynamics of four interacting neuronal populations, at the scale we are interested in, it describes the dynamics of a single node in a wider network consisting of other interacting neural masses. Following previous studies [33,48], we account for the coupling between neural masses (nodes) using the extrinsic input parameter *p*. We make the input of the *j*-th node both time and node dependent as follows,

$$p_{j}(t)=p_{0}^{\left(j\right)}+\xi^{\left(j\right)}(t)+\frac{1}{N}\sum_{i\neq j}\lambda_{ij}a_{ij}S\{z_{2}^{\left(i\right)}(t)-z_{3}^{\left(i\right)}(t)-z_{4}^{\left(i\right)}(t)\}.$$

Here the index *j* denotes node *j* (*j* = 1,2,…,*N*, where *N* is the number of nodes). $p_{0}^{\left(j\right)}$ is used to control the distance to the SNIC bifurcation; *ξ*^(*j*)^(*t*) represents noisy inputs from other areas of the cortex outside of the network under consideration; *λ*_*ij*_ is the coupling strength from node *i* to node *j*; and *a*_*ij*_ is the *i*,*j*^*th*^ entry of the adjacency matrix (the node receives the outputs of all his in-neighbours) [33,48]. We consider Gaussian noise with mean $p_{0}^{\left(j\right)}$ and

$$\langle \xi^{\left(i\right)}(t)\xi^{\left(j\right)}(t^{\prime })\rangle =\sigma_{p}^{2}\;\delta_{i,j}\delta (t-t\prime ),$$

where $\sigma_{p}^{2}$ is the variance. A node is in a resting state if *p*_*j*_(*t*) < *p*_*c*_, where *p*_*c*_ is the critical point at which the SNIC bifurcation takes place.

### Canonical model

Since the Wendling Model (WM) becomes computationally expensive for studying large networks, we look for a parsimonious representation for spiking dynamics in brain networks. Taking into account that nodes of WM are operating in the vicinity of a SNIC bifurcation, we substitute networks of neural masses with networks in which each node is represented by the normal form of the SNIC, i.e. the theta-neuron model [45]. It is important to stress that although this model is traditionally used to describe the dynamics of a neuron, here we use it (as effectively the canonical form of the SNIC bifurcation) to represent the dynamics of a neural mass in an epileptic spiking regime. The canonical model (CM) is an alternative formulation of a quadratic integrate and fire neuron. It comprises the following ODE:

$${\dot{\theta }}_{j}=(1-\cos\theta_{j})+(1+\cos\theta_{j})I_{j}(t),$$

where *θ*_*j*_ is the phase of node *j*, and *I*_*j*_(*t*) is its input current. The SNIC bifurcation occurs at *I*_*c*_ = 0. At *I*_*j*_ < *I*_*c*_, the phase oscillator is resting, whereas at *I*_*j*_ > *I*_*c*_ it is oscillating.

We define the coupling between the “canonical neural masses” analogous to the coupling defined within the WM,

$$I_{j}(t)=I_{0}^{\left(j\right)}+\xi^{\left(j\right)}(t)+\frac{1}{N}\sum_{i\neq j}w_{ij}\;a_{ij}\;[1-\cos\left(\theta_{i}-\theta_{i}^{\left(s\right)}\right)],$$

where *I*_*j*_ is the input current of node *j*, $I_{0}^{\left(j\right)}+\xi^{\left(j\right)}(t)$ represents noisy inputs coming from other areas, *w*_*ij*_ is the coupling strength from node *i* to node *j*, and *a*_*ij*_ is the *i*,*j*^*th*^ entry of the adjacency matrix. As in the WM, we consider Gaussian noise (mean $I_{0}^{\left(j\right)}$, and variance $\sigma_{I}^{2}$). We define the output of the in-neighbour *i* as $1-\cos\left(\theta_{i}-\theta_{i}^{\left(s\right)}\right)$, where $\theta_{i}^{\left(s\right)}$ is its steady state, so that if the node is resting its output is zero, and if it reaches $\theta_{i}^{\left(s\right)}+\pi$, its output is maximum. This uncoupled steady state $\theta_{i}^{\left(s\right)}$ is obtained from setting ${\dot{\theta }}_{i}=0$,

$$\theta_{i}^{\left(s\right)}=-Re\left\{\cos^{-1}\left(\frac{1+I_{0}^{\left(i\right)}}{1-I_{0}^{\left(i\right)}}\right)\right\}.$$

We take the real part so that $\theta_{i}^{\left(s\right)}=0$ at $I_{0}^{\left(i\right)}>0$. At $I_{0}^{\left(i\right)}<0$, there are two fixed points: $\theta_{i}^{\left(s\right)}$ is a stable fixed point, and $-\theta_{i}^{\left(s\right)}$ is an unstable fixed point. A similar coupling in networks of theta-neurons was recently studied in [49]. Other authors have considered delta-like interactions [50], or rapid rises in the *synaptic gating variable* [51], which are a reasonable approximation for neurons, but inappropriate for neural masses. Note that the output of a neural mass is an average over the activity of a population of neurons, and so it displays properties of a low-pass filter [52].

### Parameter comparison

For simplicity, we consider homogeneous nodes in both models, i.e., all nodes in a network are at the same distance to the SNIC bifurcation ($p_{0}^{\left(j\right)}=p_{0}$ and $I_{0}^{\left(j\right)}=I_{0}$), and have the same coupling strength (*λ*_*ij*_ = *λ* and *w*_*ij*_ = *w*). This is a strong assumption that enables us to focus explicitly on the contribution of the network structure to the network ictogenicity. Thus, there are three free parameters in each model: (*p*_0_,*σ*_*p*_,*λ*) in WM, and (*I*_0_,*σ*_*I*_,*w*) in the CM. Since our aim is to consider whether the two network models display similar changes in dynamics upon the removal of nodes, it is important that these parameters are comparable between models. Taking into account that we require that the node dynamics switch between the resting state and the spiking dynamics, the three parameters are interdependent. For example, as parameter values of the nodes move closer to the SNIC the required noise variance to elicit spikes becomes smaller. Note, however, that the variance of the noise should not be too large as we wish to ensure that network interactions play a role in the emergent dynamics. Thus, we define $\sigma_{p}^{*}=\sigma_{p}/(p_{c}-p_{0})$ and $\sigma_{I}^{*}=\sigma_{I}/(I_{c}-I_{0})$ to scale the effect of noise by the distance to the SNIC bifurcation so that the effect of the noise on the dynamics of both models is comparable. In order to establish a relation between the coupling strength and the noise, we also define λ* = 2*e*_0_*cλ*/(*Nσ*_*p*_) and w* = 2*cw*/(*Nσ*_*I*_), where *c* is the mean degree of the network. These relations compare the noise to the average maximum input that a node can receive, 2*e*_0_*cλ*/*N* and 2*cw*/*N* for WM and the CM, respectively. It provides a scale that compares noise perturbations to inputs received from in-neighbours.

Note that, with respect to the input parameter, the dynamics of a node *j* change from resting to spiking in WM if *p*_*j*_(*t*) > *p*_*c*_, and likewise, in the CM a node *j* transitions to spiking if *I*_*j*_(*t*) > *I*_*c*_. Thus, in both models we have the following condition for a node *j* to be in the parameter region corresponding to a spiking regime at time *t*,

$$x_{0}^{\left(j\right)}+\xi^{\left(j\right)}(t)+\frac{C}{N}\sum_{i\neq j}a_{ij}Y_{i}(t)>T,$$

where $x_{0}^{\left(j\right)}+\xi^{\left(j\right)}(t)$ is the noise, *C* the homogeneous coupling strength, *Y*_*i*_(*t*) the output of node *i*, and *T* the bifurcation point. If we assume that the network is in the resting state with an average node output of 〈*Y*〉, then we can estimate the critical coupling *C*_*c*_ at which on average a certain node starts to spike,

$$C_{c}=\frac{N\;(T-[x_{0}^{\left(j\right)}+\langle \xi^{\left(j\right)}(t)\rangle ])}{\langle Y\rangle k_{j}^{\left(i\right)}},$$

where $k_{j}^{\left(i\right)}$ is the in-degree of node *j* (〈*ξ*^(*j*)^(*t*)〉 = 0 in the case of Gaussian noise). Therefore, for a given network of size *N*, the larger the in-degree, the smaller is *C*_*c*_, meaning that nodes with higher in-degree are more likely to transition to spiking. This is valid in both models. Similarly, one can find the critical distance to the SNIC bifurcation,

$$x_{0c}^{\left(j\right)}=T-\frac{C\langle Y\rangle k_{j}^{\left(i\right)}}{N},$$

which is smaller than *T* due to the inputs from the network (〈*Y*〉 > 0).

### Artificial networks and measurements of nodes

The adjacency matrix encodes the network structure on top of which the nodes interact. We consider random, scale-free, small-world and rich-club networks, both directed and undirected (we discarded networks with disconnected components) [53,54]. In order to quantify the “importance” of each node, we analyze the following traditional measures: degree, average neighbour degree, eigenvector centrality, betweenness centrality, closeness centrality, clustering coefficient, and local efficiency [55,56]. Additionally, we also consider eigencentrality based on Jaccard dissimilarity [57] and dynamical importance [58]. In the case of directed networks, we also consider in-degree, out-degree, as well as the sum and product of these measures.

### Definition of spiking dynamics and treatment perturbations

We focus our analysis upon two measurements that are relevant for our purposes of studying epileptic dynamics and surgery *in silico*, namely *Brain Network Ictogenicity* (*BNI*) [23,42,59], and *Node Ictogenicity* (*NI*) [42]. *BNI* is a practical approach for quantifying the tendency of a network to generate spiking dynamics. It measures the average fraction of time spent in spiking dynamics by each node [23,42,59]:

$$BNI=\frac{1}{N}\sum_{i}\frac{Time\;spent\;in\;spiking\;dynamics\;by\;node\;i}{Total\;time}.$$

Specifically, in the WM, first we extract the spikes generated by a node by applying a threshold to the average absolute amplitude of the model output over a sliding window of 0.05 s. Then, contiguous epochs of spiking dynamics are identified by evaluating the overlap of 1 s time windows centred in each spike. Finally, the time spent in spiking dynamics corresponds to the total time of these spiking epochs [42]. In the CM we use the same method, with similar time scales (we use as conversion time scale the ratio of the full widths at half maximum of the spikes in each model).

*NI* quantifies the contribution of each node to the ictogenicity of the network by measuring the relative difference in *BNI* upon removing node *i* from the network:

$$NI^{i}=\frac{BNI_{pre}-BNI_{post}^{i}}{BNI_{pre}},$$

where *BNI*_*pre*_ corresponds to the *BNI* over the network prior to node resection and $BNI_{post}^{i}$ is the *BNI* after the removal of node *i*. Note that *NI*^*i*^ = 1 means that the removal of node *i* renders the network free of spiking dynamics, whereas *NI*^*i*^ = 0 means that the resection of node *i* made no difference to the *BNI*. In practice, this quantity measures the success of a given surgery resection *in silico*, and it may have the potential to guide the search for an optimal surgical strategy. In general, this quantity may also be useful to quantify the result of temporary ablation, assuming that the ablation takes place in a much slower time scale than the network dynamics. In this paper we set *BNI*_*pre*_ = 0.5 (we have confirmed that the results are qualitatively the same for other reference values of *BNI*_*pre*_).

To evaluate if the CM can be used as a proxy of the WM in this framework, we compare the *NI* ordering of the two models for a number of networks. Note that *NI* is essentially a vector with *N* entries quantifying the result of removing each node individually, being of particular interest the relative impact of each node removal compared to the others, rather than the absolute value of each one (which is parameter dependent). We use a weighted Kendall's rank correlation measure [60,61], which is defined as follows. Given two rankings (*NI*) of the same items (nodes of the network), we calculate

$$\tau =\frac{P-Q}{P+Q},$$

where *P* is the number of items in the same order in the two rankings, and *Q* counts the number of items in reverse order. When *τ* = 1 the two rankings predict the same ordering, whereas *τ* = −1 means a reverse order of all items. Here we consider a weighted measure to take into account the relative values of *NI*: each *NI* comparison between two nodes *i* and *j* is weighted by the product of the distances in *NI* predicted by the two models, $\left|NI_{WM}^{i}-NI_{WM}^{j}|\times |NI_{CM}^{i}-NI_{CM}^{j}\right|$, (where $NI_{WM}^{i}$ and $NI_{CM}^{i}$ are the *NI*s of node *i* calculated using WM and the CM, respectively). We assume that there are no ties.

### Patient data and functional networks

We focus on patients with pharmacoresistant epilepsy, since such patients are candidates for surgery. Data were collected from 16 patients (11 female, mean age 31, and median post-surgical follow up 3 years) who underwent pre-surgical monitoring at Inselspital Bern [42,62]. Following epilepsy surgery, six patients fell into Engel class I (free of disabling seizures), five into Engel class II (rare disabling seizures) and five into Engel class IV (no worthwhile improvement). All patients gave written informed consent that imaging and iEEG data may be used for research purposes. Other details about the data can be found elsewhere [42,62]. Before analysis, the signals were down-sampled to a sampling rate of 512 Hz and re-referenced against the median of all the channels free of permanent artefacts as judged by visual inspection by an experienced epileptologist (K.S.). For each patient, two peri-ictal epochs were considered, which included three minutes before seizure onset, the seizure itself and three minutes after seizure termination (seizure onset and offset were identified by visual inspection (K.S.)). Following the methods described in [62,63], first we applied a band-pass filter between 0.5 and 120 Hz and a notch filter (48 to 52 Hz) using a Butterworth filter. Each epoch was divided in a set of 8 seconds segments (the segments were chosen 1 second apart from each other). For each segment we obtained 10 univariate iterated amplitude adjusted Fourier transform (IAAFT) surrogates independently. Next, the segments were divided in 10 subsegments of 1024 sampling points (2 seconds) distributed with minimal overlap. Thus, we generated an ensemble of 10 subsegments for the original time series, and 100 subsegments for the surrogates (10 for each surrogate). To estimate the correlations between the time series of each iEEG channel, we used the Pearson's equal-time (zero-lag) cross-correlation coefficient *ρ*, and a non-parametric Mann-Whitney-Wilcoxon U-test was performed to assess the significance of different medians of *ρ* between the original time series (*ρ*_*o*_) and the surrogates (*ρ*_*surr*_). We further applied Bonferroni-Holm corrections to account for multiple comparisons. Finally, we obtained a surrogate-corrected correlation matrix using the heuristic formula [63,64]

$$C=\frac{\rho_{o}-\rho_{surr}}{1-\rho_{surr}}s,$$

where *s* = 1 if the null hypothesis of the statistical test is rejected, or *s* = 0 otherwise. Using this method, we derived 102 ± 18 functional networks based on cross-correlation for each patient, depending on the duration of each seizure epoch.

### Rich-club organization

The organization of functionally derived networks into rich-clubs [65–67] was studied using a weighted rich-club parameter *ϕ*(*k*) [66]. The richness parameter is the degree *k*, and the procedure consists in finding groups (clubs) of nodes whose richness is larger than *k*. For a given degree *k*, we counted the number of connections *E*_>*k*_ of the club, and summed their weights *W*_>*k*_. We then calculated the fraction of weights shared by the club out of the maximum edge weights that the club could have if they were linked by the strongest connections of the network, i.e.,

$$\phi^{w}(k)=\frac{W_{>k}}{\sum_{l=1}^{E_{>k}}w_{l}^{rank}},$$

where $w_{l}^{rank}$ are the ranked weights of the network. This fraction is not enough to verify the existence of a rich-club, since even random networks can have an increasing function *ϕ*^*w*^(*k*) as a result of chance alone (nodes with higher degree are more likely to be connected). Therefore, *ϕ*^*w*^(*k*) is normalized relative to *ϕ*^*rand*^(*k*) obtained from a set of comparable random networks,

$$\phi (k)=\frac{\phi^{w}(k)}{\phi^{rand}(k)}.$$

Thus, a network exhibits rich-club organization if there is a range of degree *k* for which *ϕ*(*k*) > 1 [65,66]. We generated 100 random networks by applying a reshuffle procedure to the weights while keeping the topology of the original network intact, followed by a link and weight reshuffle procedure that preserves the original degree distribution [56,67]. *ϕ*^*rand*^(*k*) was calculated as the average rich-club coefficient for each level of *k*. Finally, we evaluate the statistical significance of rich-club organization using a permutation test [67], by testing whether *ϕ*(*k*) was statistically significantly larger than *ϕ*^*rand*^(*k*) (a one-sided *p* value was calculated as the percentage of the distribution of *ϕ*^*rand*^(*k*) that exceeds *ϕ*(*k*)). We measured the rich-clubs of the average functional networks of the pre-seizure, seizure, post-seizure, and whole peri-ictal epochs for each patient separately.

## Results

### Predictions for the effect of node removals are preserved in a canonical model

**Fig 1:**
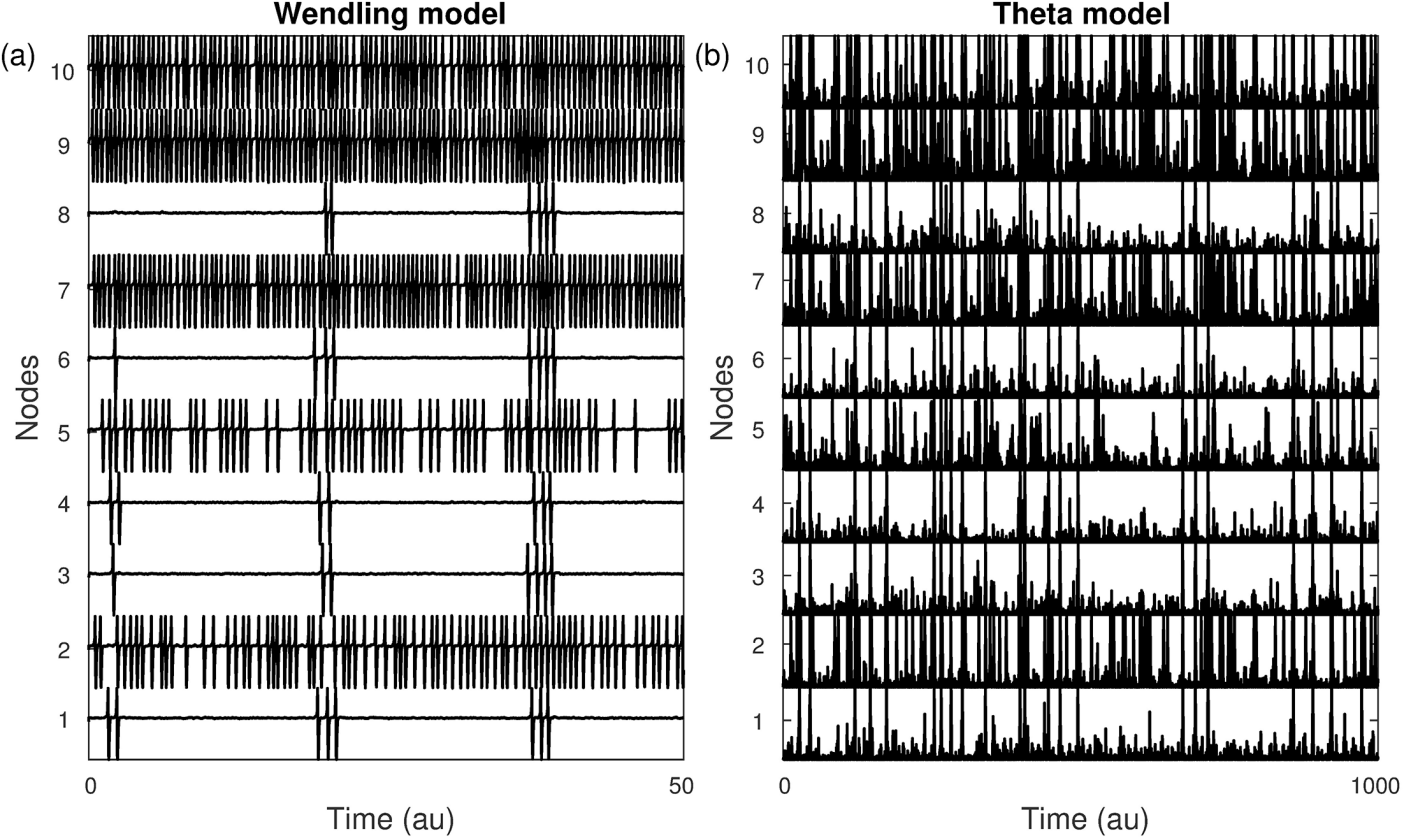
Node activities in the (a) WM and (b) CM. Although the spikes have different forms between models and the CM shows higher noisy activity, both models identify the same set of nodes as having a larger propensity to spike. The network is the same for both models, a directed random network with *N* = 10 nodes and mean degree *c* = 1.6. Parameters: $\left(p_{0},\sigma_{p}^{*},\lambda^{*})=(94,5,2.57\right)$ and $\left(I,\sigma_{I}^{*},w^{*})=(-1.2,5,1.6\right)$.

We compared the dynamics of the CM to the WM in terms of the effect that model parameters have on *BNI* and the profile of *NI* for a suite of networks. Fig 1 demonstrates typical dynamics of each model applied to the same network (a directed random network with *N* = 10, and mean degree *c* = 1.6). Both models display spiking dynamics, with a heterogeneous distribution of activity across nodes. For each model, nodes 2, 5, 7, 9 and 10 show a greater extent of spiking dynamics than other nodes; thus the distribution of activity across the network is preserved in the canonical model. On the other hand, it is clear that the resting state is noisier in the CM. A predominant feature accounting for this is that the ratio of amplitude of the spiking trajectory to noise is larger in the WM. Moreover, one should also realize that whereas in the WM only positive inputs can move the system towards the SNIC bifurcation, in the CM both positive and negative inputs displace the phase, *θ*, from the resting state. Furthermore, the output of the resting state in the CM is zero, but non-zero in the WM.

**Fig 2:**
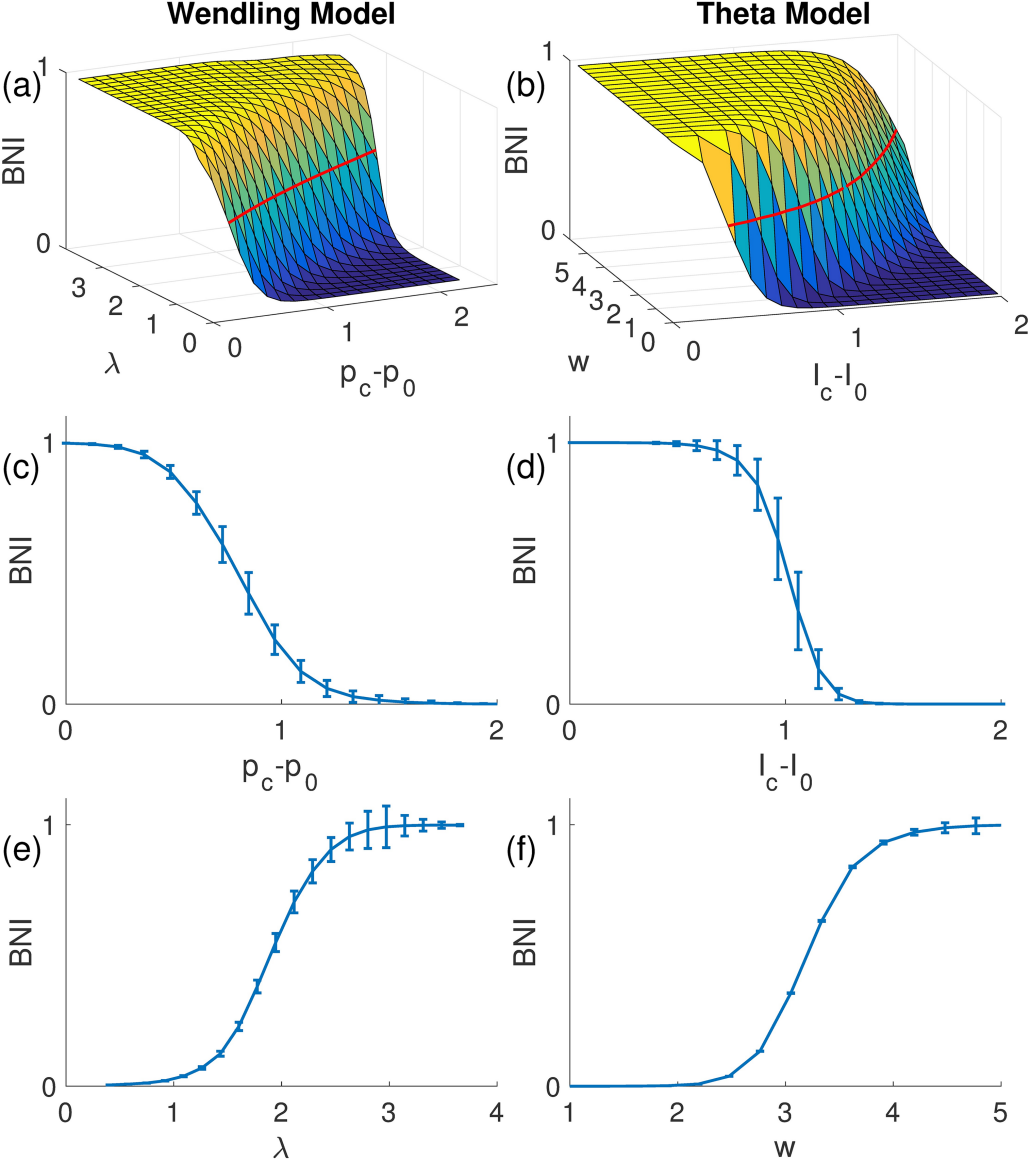
*BNI* as function of coupling strength and distance to the SNIC bifurcation. The left and right columns correspond to the WM and CM, respectively. The resemblance between the two surfaces suggests that the CM is a good parsimonious model of WM for the of study *BNI*. The red lines on the surfaces correspond to *BNI*_*pre*_ = 0.5. The surfaces in panels (a) and (b) represent an average over an ensemble of 10 directed random networks, and the error bars in panels (c)-(f) account for the variability of *BNI* between different network realizations. Parameters: $\sigma_{p}^{*}=\sigma_{I}^{*}=5,\;N=15$, and *c* = 1.6. Additionally, in panels (c) λ* = 1.09, (d) w* = 1.33, (e) *p*_*c*_−*p*_0_ = 1.45, and (f) *I*_*c*_−*I*_0_ = 1.62.

As described in the Methods, although we have identified three free parameters, the network dynamics are in fact affected by only two competing factors: the distance to the SNIC bifurcation and the coupling strength. The strength of noise required to elicit spikes is correlated with the distance to the SNIC bifurcation (that is smaller noise variance is required to elicit spikes the closer the system is to the bifurcation). Thus, we can fix the noise variance and consider *BNI* as a function of the distance to the SNIC bifurcation and coupling strength. Fig 2 provides an evaluation of this function for an ensemble of 10 random networks with 15 nodes and demonstrates that the smaller the distance to the bifurcation, the easier it is to generate spiking dynamics, and consequently *BNI* is larger. In addition, *BNI* grows with increases in coupling strength. Fig 2 demonstrates that the shape of the *BNI* surface is similar for the two models, which provides evidence that the normal form of the SNIC is appropriate for the study of the propensity of a network to generate spiking dynamics. Similar results were obtained for both smaller and larger networks.

**Fig 3:**
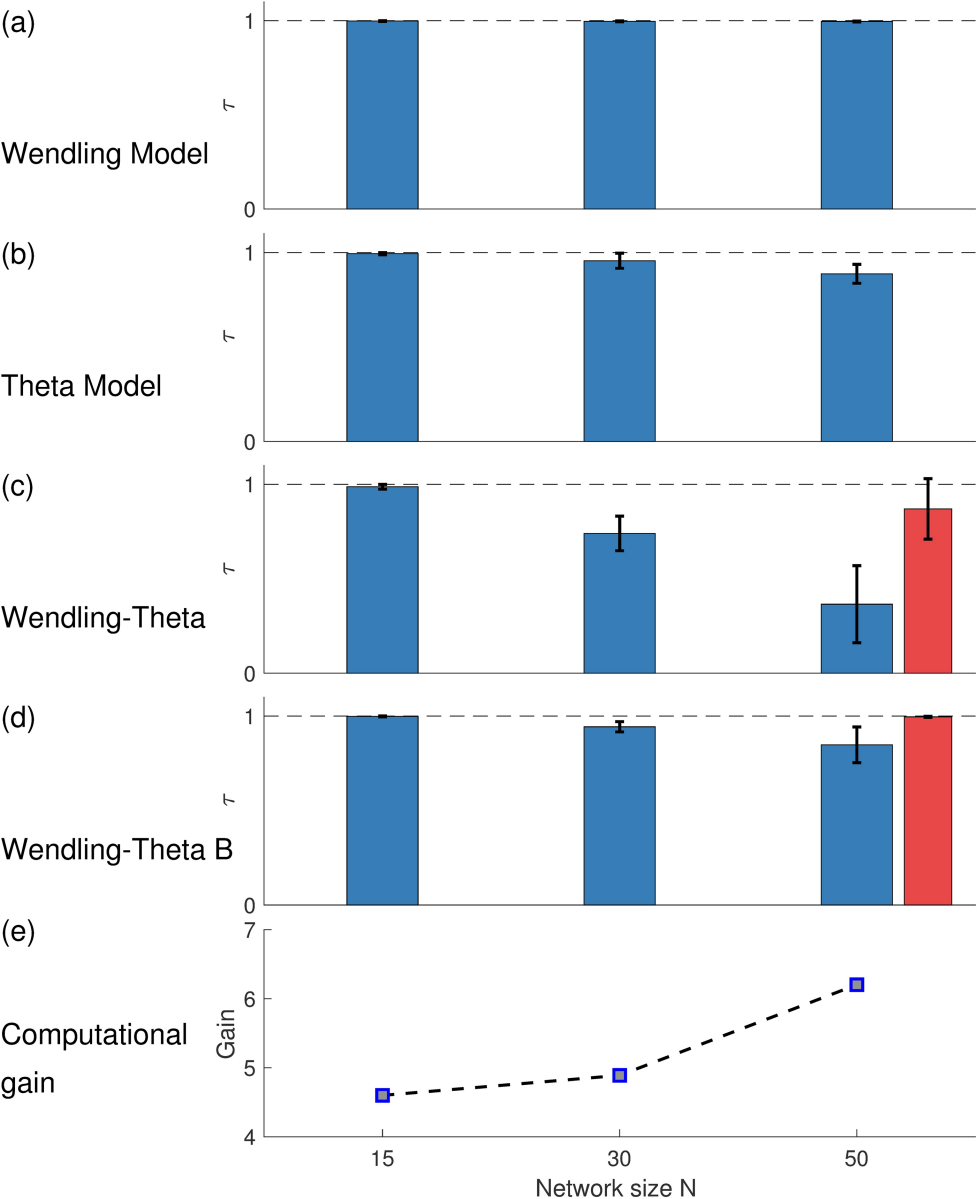
Consistency of *NI* within and between models and computational gain. The consistency of ordering nodes according to their *NI* value, quantified by Kendall correlation rank *τ* within (a) the WM, (b) the CM, and (c),(d) comparison between the two models. The comparison is performed for networks of size *N* = 15, 30 and 50. In each scenario 10 realizations of directed random networks with mean degree *c* = 1.6, 3.2, and 5.3 are considered. For each case, the *NI* ranking is contrasted across 5 different parameter realizations. In panel (c) all combinations of parameters are compared, whereas in panel (d) the parameter set that maximizes *τ* is used. Although the distribution of *NI* tends to differ between the models as *N* increases, panel (d) shows that it is possible to find parameters such that the *NI* ordering is consistent between models. The red bars in panels (c) and (d) correspond to undirected scale-free networks (10 network realizations, *N* = 50, *γ* = 2, and *c* = 6). Panel (e) demonstrates the computational gain of using the CM relative to the WM. The gain corresponds to the computational time the WM takes to compute the *BNI* divided by the time the CM takes to perform the same task.

**Fig 4:**
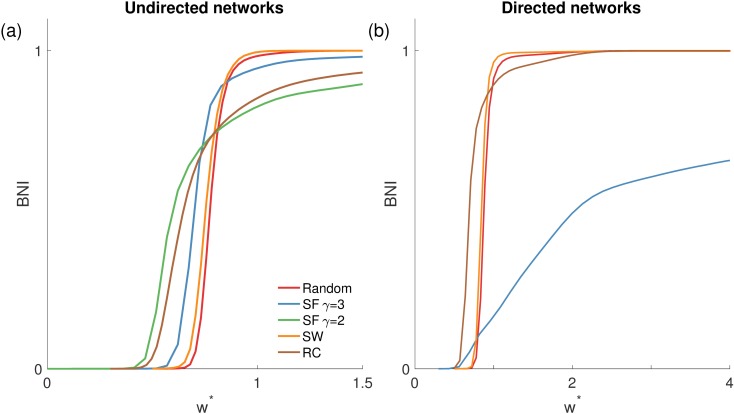
*BNI*(w*) for different network structures in the CM, namely in panel (a) undirected and in panel (b) directed networks. The red curves correspond to random networks, the blue and green to scale-free (SF) networks with *γ* = 3 or *γ* = 2, respectively, the orange to small-world (SW) networks, and the brown to rich-club (RC) networks. Note that scale-free and rich-club networks show a non-zero *BNI* value at smaller coupling strengths, and take higher strengths to get to *BNI* = 1 in comparison to random and small-world networks. Each curve is an average over 10 network realizations of the same topology. Parameters: *N* = 64, *c* = 6, probability of rewiring for SW networks *p* = 0.5, and the rich-club has 10 nodes (with *p*_1_ = 0.7, and *p*_2_ = 0.2 the connection probabilities within and from the rich-club to the rest of the network, respectively).

Our results thus far indicate that despite some expected quantitative differences, network dynamics, and in particular the way that *BNI* changes with respect to system parameters, are qualitatively similar across the WM and the CM. However, our primary focus is to determine whether the CM would provide the same prediction for the effect on *BNI* of node removals (i.e. *NI*). With the application of surgical resections in mind, we are predominantly interested in how comparable the ordering of *NI* is between the two models. In order to investigate this, we calculated the distribution of *NI* for a suite of random networks of size *N* = 15,30 and 50 and calculated the similarity in ordering of *NI* using Kendall's *τ* (see Methods). Fig 3 shows that within models, the *NI* distribution is robust across different parameter sets for which *BNI*_*pre*_ = 0.5, which is our starting point for the calculation of *NI* (see Methods) and defines a line in the surface of Fig 2. Across different choices of parameters within the WM, we find *τ* > 0.97 for all networks considered, indicating a strong preservation of the ordering of *NI* when different parameters are used. Within the CM, *τ* > 0.89 and thus there is slightly more variation across *NI* orderings for this model. Fig 3C and 3D show *τ* for comparisons of *NI* orderings between the two models. Fig 3C demonstrates that when model parameters are chosen randomly, the ordering of *NI* is preserved between the two models for small networks, but differences in predictions between the two models arise in larger networks (for example with 50 nodes). However, Fig 4D demonstrates that a parameter set for each model can be found such that *NI* distributions are preserved across models in the larger networks studied (50 nodes, *τ* = 0.85 ± 0.09).

We note that as *N* increases, nodes become topologically similar in a random network, and therefore one can expect a homogeneous distribution of *NI*. However, we are primarily interested in networks for which nodes exist that should be resected to reduce the presence of spiking dynamics. We therefore study networks for which we might expect the distribution of *NI* to be heterogeneous (as we will show below). A natural choice is a scale-free network characterized by a power law degree distribution *P*(*k*)∼*k*^−*γ*^ with a small exponent (*γ* < 3) [54]. Fig 3 demonstrates that for scale-free networks arbitrary choices of parameters yield a strong similarity in ordering of *NI* (*τ* = 0.87 ± 0.16) and that model parameters exist for which the ordering is essentially identical (*τ* = 0.996 ± 0.003).

Fig 3E demonstrates the computational advantage gained by using the CM over the WM. We find that the ratio of computational time of the WM to the CM when estimating *BNI* is 4.6 for networks of size *N* = 15, 4.9 for *N* = 30, and 6.2 for *N* = 50. Note that this gain does not correspond to the ratio of floating point operations needed by each model to simulate a time step because the time scales are different between these two models. Crucially, such gain will be very useful when applying this framework in the clinical setting, as it represents a speed-up in the computational time from days to hours.

Having demonstrated similarity in the ordering of *NI* across the WM and CM, we proceed in the following sections to use the CM to study how *NI* varies across different types of network. We fix the number of nodes that we consider to be 64, in line with a typical number of iEEG and depth electrodes used in pre-surgical planning applications [42,62,68].

### The role of topology in network ictogenicity

Fig 4 shows how *BNI* varies as a function of the coupling strength under different choices of network topology. Scale-free networks are the most prone to transit to spiking dynamics since *BNI* becomes non-zero for smaller coupling strengths relative to the other topologies. The effect is more noticeable in networks with smaller exponent *γ*, which have a greater degree variance (i.e. are more heterogeneous). However, the maximal value of *BNI* is less than one for scale-free networks, in particular in directed networks. Fig 4 demonstrates that rich-club networks exhibit a similar profile of increases in *BNI* with increases coupling strength features to scale-free networks, which is presumably a consequence of similarities in the degree distributions of these networks. Small-world and random networks have similar profiles, implying that the high clustering coefficient of small-world networks has little impact on a network’s ictogenicity (*BNI*). Similar results were obtained for smaller and larger networks (up to *N* = 128), as well as for sparser (*c* = 4) and denser networks (*c* = 10), and in the case of small-world networks for smaller rewiring probabilities (*p* = 0.1). Fig 4B shows that whilst the profile of random and small-world networks is similar for directed and undirected networks, the profile changes for rich-club and scale-free networks, most significantly for scale-free networks, whose *BNI* in directed networks has a very gradual increase with increasing coupling strength. This is likely due to the disparity between the in- and out-degree of nodes (note that nodes can have a high out-degree but a low in-degree, meaning they can influence the network activity, but not be influenced by it, and vice versa).

### The role of topology in node ictogenicity

**Fig 5:**
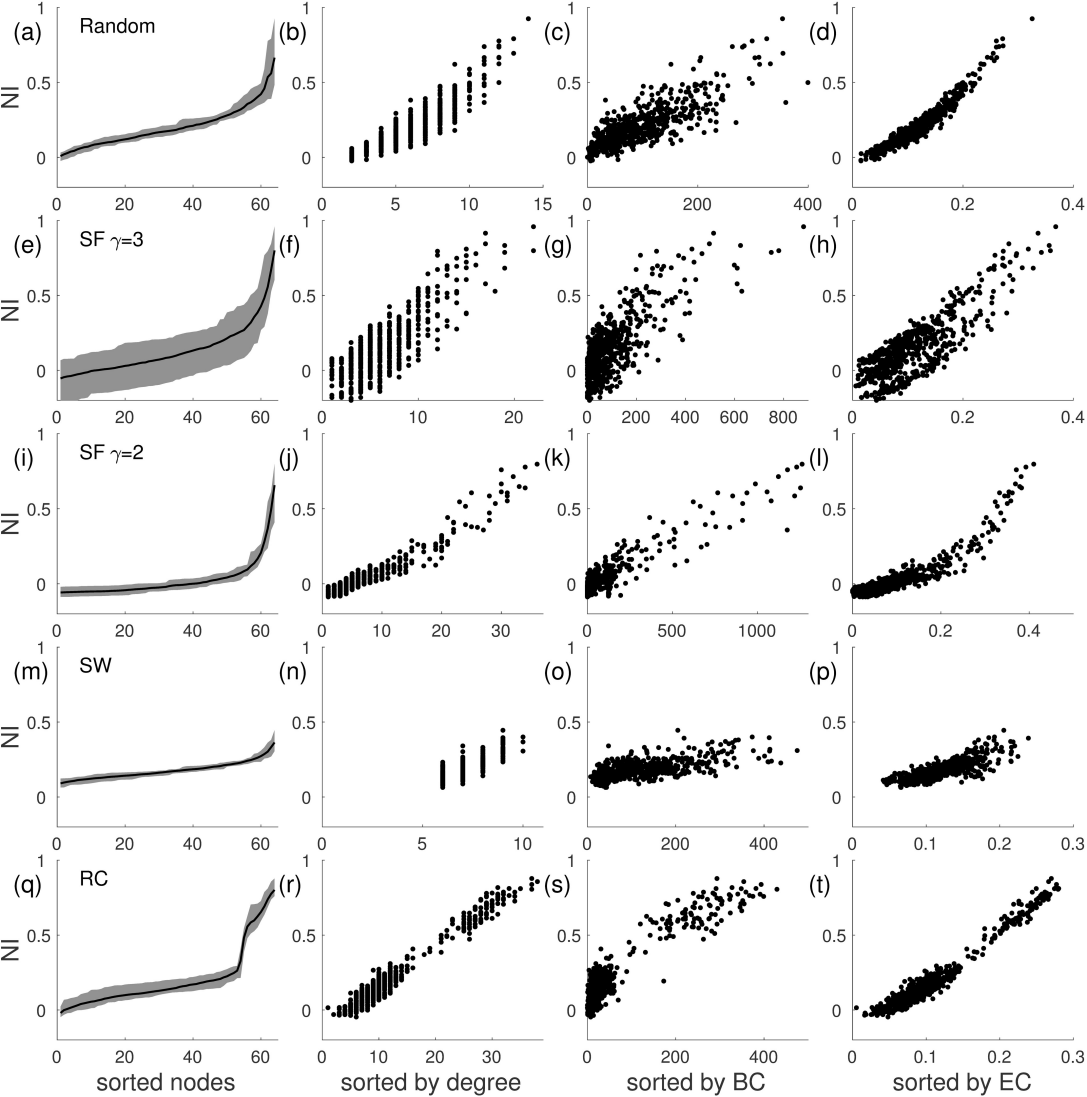
Comparison of *NI* across different undirected network structures and its relation to node properties in the CM. Each row corresponds to a different network topology: (a)-(d) random network; (e)-(h) scale-free network (SF) (*γ* = 3); (i)-(l) scale-free network (*γ* = 2); (m)-(p) small-world network (SW); (q)-(t) rich-club (RC) network. In the first column the nodes are sorted by their *NI*, so that it is monotonically increasing; in the second column *NI* is sorted by the degree of the nodes; in the third column it is sorted by the betweenness centrality (BC); and in the fourth column it is sorted by the eigenvector centrality (EC). A linear correlation is particularly clear between *NI* and eigenvector centrality. The shaded areas in the first column and the dots in the other panels correspond to 10 different network realizations of the same topology (the line represent the mean). Parameter choices are as in Fig 4.

**Fig 6:**
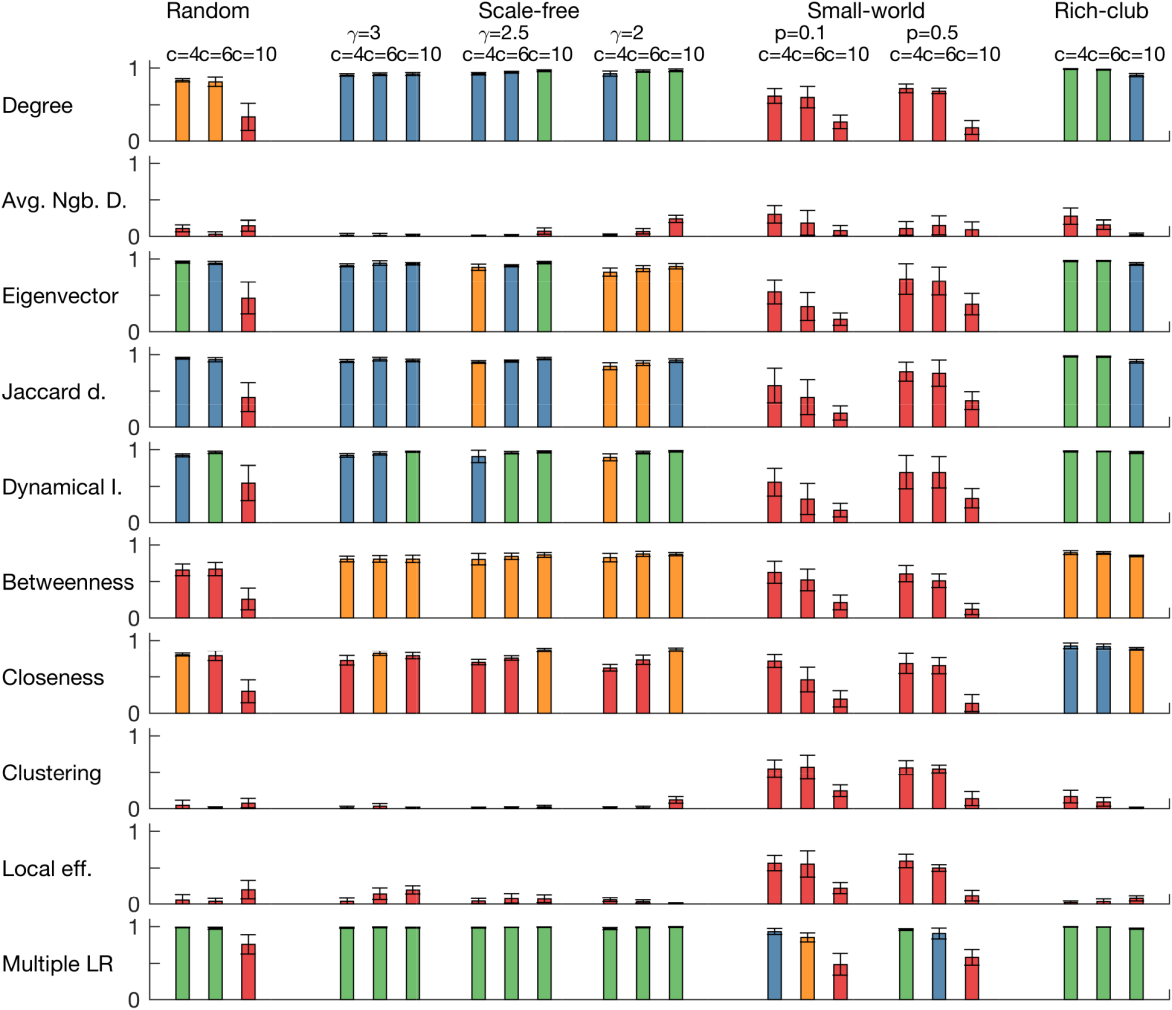
Coefficient of determination (*R*^2^) of linear regressions of *NI* for a set of node measurements applied in different network topologies for the CM. Each row corresponds to a node measurement (degree, average neighbour degree, eigenvector centrality, eigencentrality based on Jaccard dissimilarity, dynamical importance, betweeness centrality, closeness centrality, clustering coefficient, and local efficiency), whereas each column corresponds to a different network topology (within topologies, we present results for three different mean degrees). The last row is the *R*^2^ of a multiple linear regression combining all measurements. Eigenvector centrality, eigencentrality based on Jaccard dissimilarity, and dynamical importance are the node measures whose correlation with *NI* is higher. Colour code: green corresponds to *R*^2^ > 0.95; blue to 0.90 < *R*^2^ ≤ 0.95; orange to 0.80 < *R*^2^ ≤ 0.90; and red to *R*^2^ ≤ 0.80. All networks are undirected and parameter choices are as in Fig 4.

Fig 5 demonstrates the way that *NI* is distributed amongst nodes in networks with different topologies, and furthermore how *NI* correlates with graph theoretical properties of nodes. The first column of Fig 5 shows that random, scale-free and rich-club networks each have a skewed *NI* distribution, with a small subset of nodes having large *NI*, whereas small-world networks have a flat distribution, with small values of *NI* across nodes. Nodes in rich-clubs were found to have high *NI*. Similarly, we found several measures of node importance to correlate with *NI*, but in a topology-specific way. For example, whilst node degree correlates to a great extent with *NI* in scale-free networks, it does not for random networks, particularly when the mean degree is large. Eigenvector centrality and dynamical importance were found to be good predictors of *NI* (*R*^2^ > 0.80, see Fig 6) in all networks except those with small-world topology and random networks with large mean degree (*c* = 10). We found that small-world networks required all considered measures to achieve an adequate prediction of *NI* (*R*^2^ > 0.85 for multiple regressions with all the considered node properties, for *c* = 4 and *c* = 6). We note that directed networks typically did not contain nodes with *NI* > 0.5 and furthermore that *NI* did not correlate with graph theoretical measures in directed networks (*R*^2^ < 0.3, see S1 Fig). We tested the robustness of these results for other reference values of *BNI*_*pre*_ (*BNI*_*pre*_ = 0.3 and *BNI*_*pre*_ = 0.7) in all the networks (*c* = 6). We obtain similar results, although the networks become less sensible to perturbations for *BNI*_*pre*_ = 0.7.

### Targeted and random node removal

**Fig 7:**
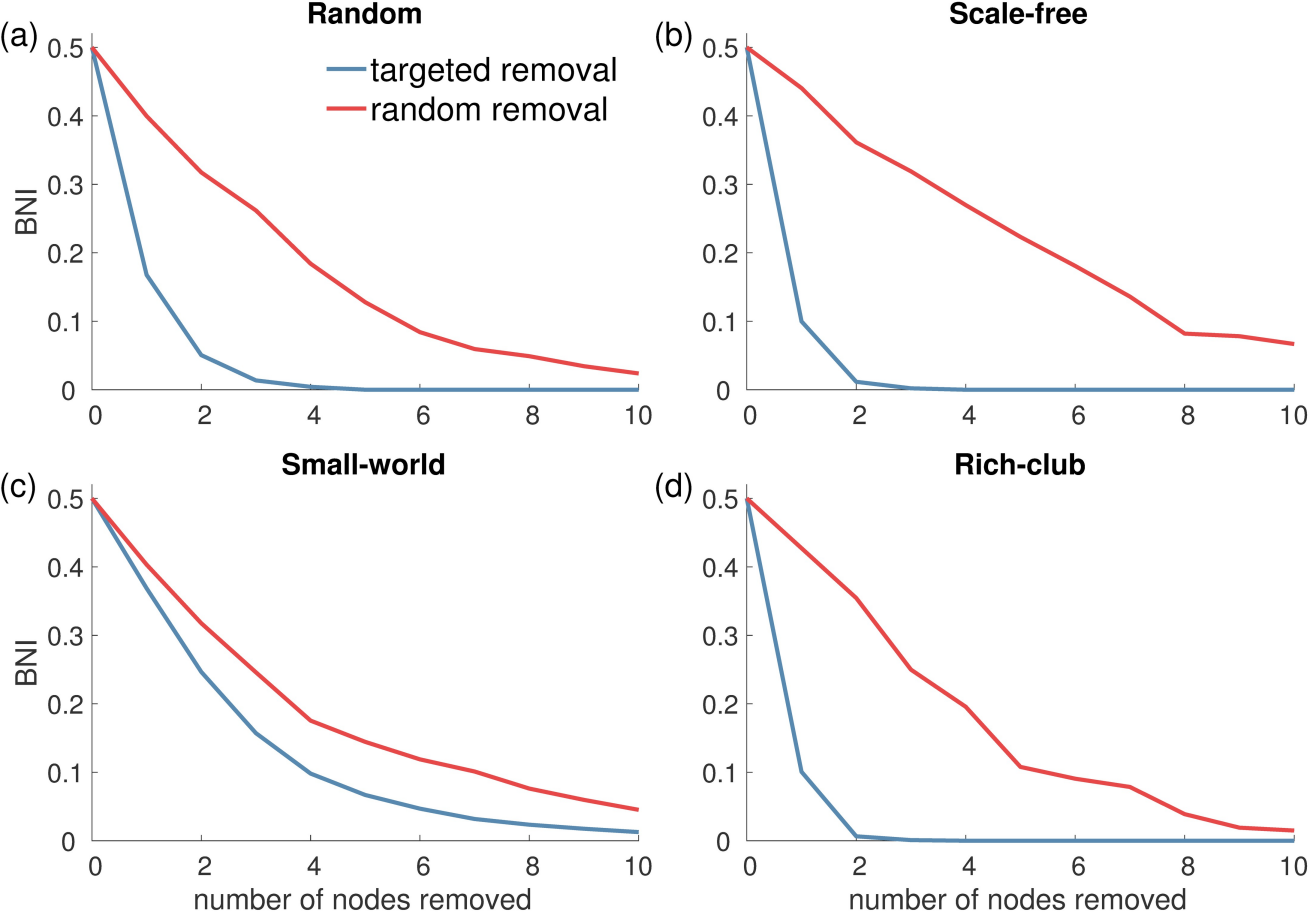
*BNI* as function of the number of nodes removed from a network in the CM. Each panel corresponds to a different undirected network topology: (a) random networks, (b) scale-free networks (*γ* = 3), (c) small-world networks, and (d) rich-club networks. The two curves represent two strategies of node removal: the blue curves correspond to targeted node removal according to highest eigenvector centrality, whereas the red curves correspond to random node removal. Target node removal is much more effective to reduce *BNI* in all network topologies except for small-world networks in which both strategies provide a similar outcome. The probability of rewiring for SW networks was *p* = 0.1, and other parameters choices were as in Fig 4.

Depending upon the particular choice of network representation, resections from brain networks could include more than one node. We therefore sought to gain insight into how many nodes have to be removed from a network in order to render it incapable of generating spiking dynamics. In order to resect a minimum number of nodes while reducing *BNI* as much as possible, it seems sensible to target the highest *NI* nodes first. Fig 7 demonstrates how this strategy compares to random node removal (we use the eigenvector centrality as a proxy of *NI* and therefore we target nodes with highest eigenvector centrality, but similar results were obtained targeting nodes with highest *NI*). Note that in the case of targeted node removal, when one node is removed, the whole network changes, and therefore the distribution of *NI* may change as well. Therefore, we recalculated the eigenvector centrality of each node of the new network after each node removal. The figure shows that a targeted node removal is much more effective than a random strategy in all topologies except small-world networks, in which the two strategies give similar outcomes. This is to be expected taking into account the highly homogenous distribution of *NI* in small-world networks (see Fig 5). Accordingly, the difference between the two strategies is particularly noticeable in scale-free networks, because of its heterogeneous *NI* distribution.

### Rich-club organization in iEEG-derived functional networks

**Fig 8:**
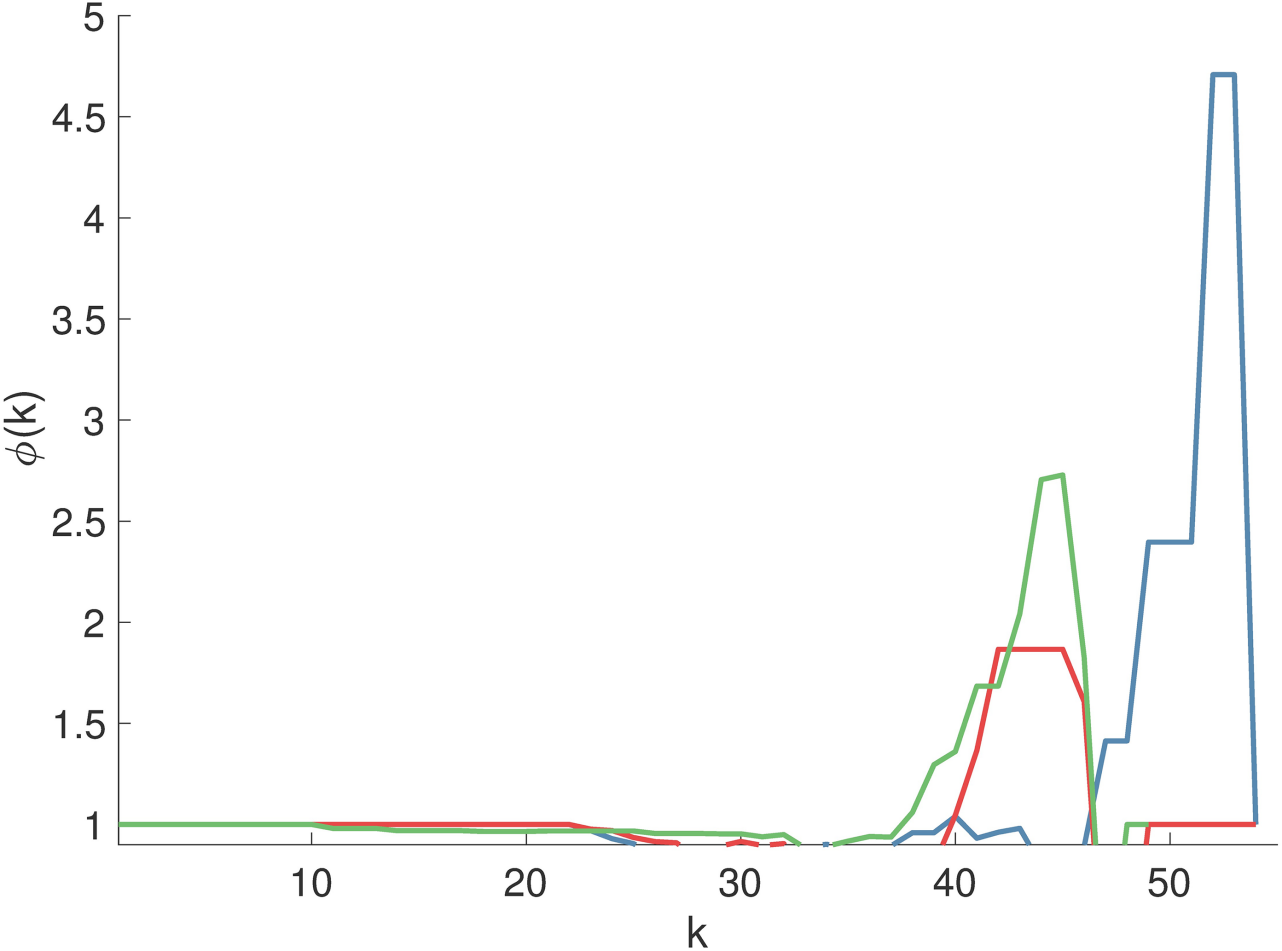
Rich-club functions *ϕ*(*k*) of functional networks of pharmacoresistant epileptic patients. Each curve *ϕ*(*k*) was obtained from an average functional network of a different patient. *ϕ*(*k*) > 1 indicates the presence of rich-club organization.

Our results thus far demonstrate the distribution of *NI* throughout a network is dependent upon network structure. In particular, rich-club, or networks with highly connected hubs were found to contain nodes with high *NI*, even though all nodes were equivalently parameterized and therefore those nodes were not apparently pathological. In a practical setting those nodes would be natural targets for epilepsy surgery. We therefore sought to understand whether typically used clinical data would yield network representations of the brain with these properties. We thus quantified the presence of rich-club organization in functional networks derived from iEEG recordings from patients that were considered for epilepsy surgery. We considered peri-ictal recordings, and we found evidence of rich-club structure in the functional networks of pre-seizure, seizure and post-seizure epochs in each patient. Fig 8 shows rich-club functions for 3 representative functional networks from seizure epochs of 3 different patients. A rich-club coefficient larger than one over a range of degree *k* indicates the presence of rich-club organization [65,66] and we found this to be the case in all patients. Note that scale-free networks also display rich-club organization [65], and so both types of network are identified by this type of analysis.

### Disruption of rich-club predicts postsurgical outcome

**Fig 9:**
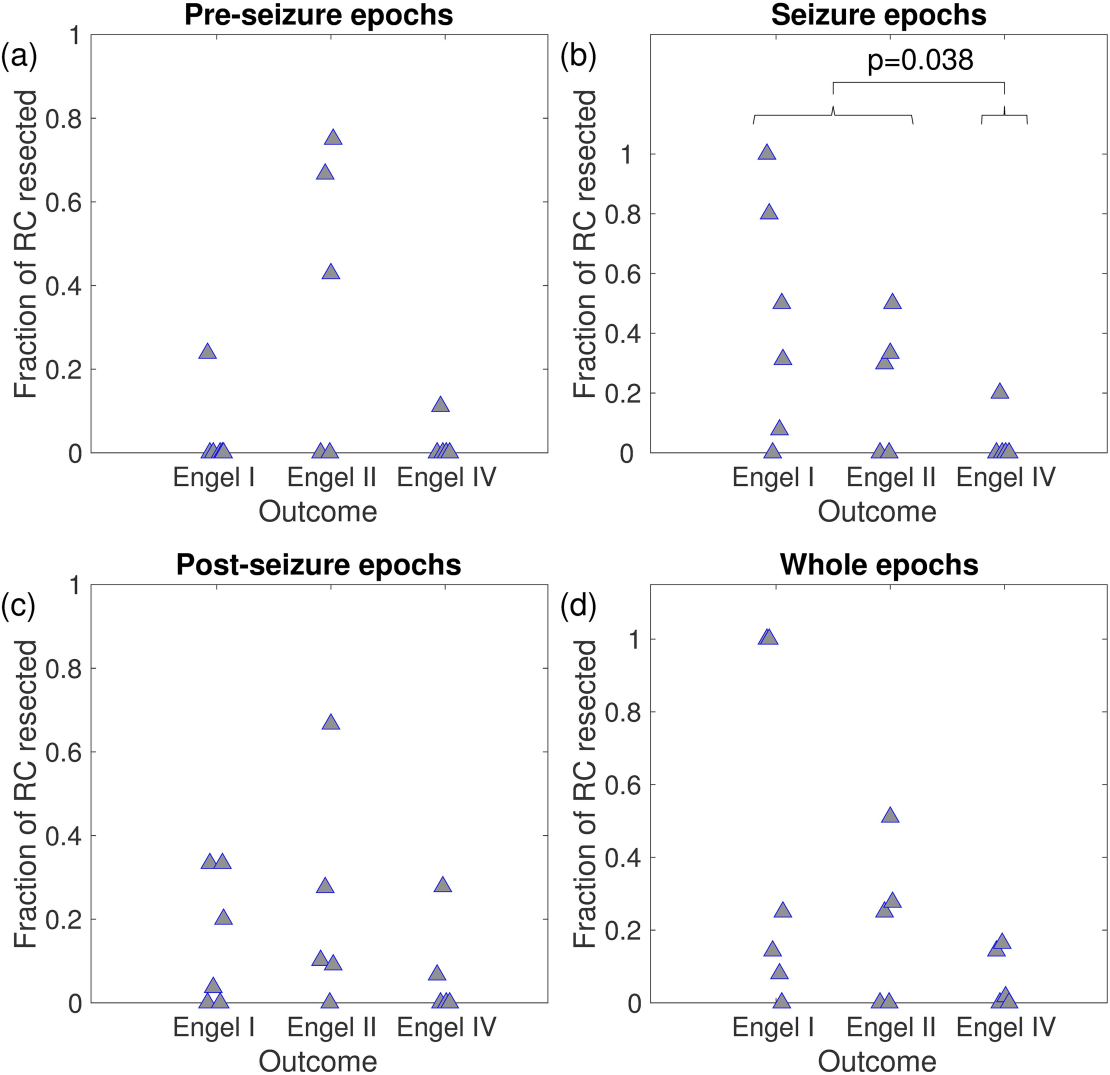
Fraction of rich-club (RC) resected versus seizure outcome for 16 patients. Panels (a), (b), (c), and (d) correspond to a quantification based on functional networks derived from pre-seizure, seizure, post-seizure, and whole epochs, respectively. Braces indicate the p-value of Kruskal-Wallis one-way analysis of variance to quantify the statistical difference between good and bad responders.

Next we extended this analysis taking into account the location of resections relative to the placement of iEEG electrodes and the postoperative outcome [42,62]. Our model analysis led us to hypothesise that if the rich-club was partially or totally resected, the outcome for patients would likely be favourable, since we would expect nodes in the rich-club to have high *NI*. To test this, we estimated which nodes were members of the rich-club in each functional network (over the pre-seizure, seizure, and post-seizure epochs and all combined) as the collection of nodes belonging to the ‘richest’ club, i.e., the nodes with degree larger than *k*_*r*_, where *k*_*r*_ corresponds to the maximum of *ϕ*(*k*) (see Methods). Fig 9 demonstrates that for networks derived from pre-seizure, post-seizure, or full peri-ictal epochs, the fraction of rich-club resected did not correlate with the outcome of surgery. However, in functional networks derived from the seizure epoch, there was a significant difference in fraction of rich-club resected between patients with good (Engel classes I and II) and poor (Engel class IV) post-operative outcome (*p* = 0.038, Kruskal-Wallis test). Patients with good postoperative outcome (seizure free or almost seizure free) had a significantly larger disruption to the rich-club than those with no postoperative improvement.

## Discussion

In this study we used a canonical form of the Wendling model to systematically explore the influence of network topology on the generation of spiking dynamics and the effect that removing nodes from a network has on its ability to generate such dynamics (i.e. its ictogenicity). We demonstrated that networks with scale-free and rich-club topology are more ictogenic in the sense that they require a smaller coupling strength between connected nodes to lead to the onset of spiking dynamics. Furthermore, we showed that on the whole, the ictogenicity of nodes within an undirected network correlate with graph theoretical measures most notably degree, eigenvector centrality and dynamical importance [58]. This led us to hypothesise that disruption of rich-clubs in networks should lead to diminished ictogenicity. We tested this hypothesis by first demonstrating the presence of rich-clubs in functional networks derived from iEEG data of people with pharmacoresistant epilepsy, and further showing a significantly greater extent of disruption to rich-club structures in patients who had good postoperative outcome, compared to those with poor postoperative outcome.

It has recently been suggested that there exists a local pathological hub near the epileptic focus responsible for spreading epileptic activity, which should be resected by surgery [69]. This hypothesis is supported by evidence demonstrating that betweenness centrality correlates with resected cortical regions in patients who had a favourable surgery outcome [70,71]. Our results are in agreement with this hypothesis. Indeed, the rich-club comprises a group of nodes with high betweenness centrality that can work as pathological hub spreading epileptic activity.

Functional networks can be thought of as representing communication pathways in the brain that are active, or open, at a given moment. In the case of brain disorders such as epilepsy, we assume that there exist pathological pathways that support the emergence of seizures (i.e. ictogenic networks). Since it is natural to assume that these networks are also expressed in electrographic data and can be characterized in terms of functional connectivity, we use such networks in our models. This is in contrast to modeling based on the structural connectivity of the brain [73]. Structural networks place a constraint on the rhythms of the brain that can emerge. However, network models of brain rhythms built on structural networks typically neglect biochemical processes taking place over multiple different time scales preventing us from knowing, at any given time, which connections are actually active. That is why we have chosen functional connectivity based modeling in this study. A potential limitation of this approach, however, is that the network studied depends on the choice of epoch and the method used to characterize functional connectivity. Interestingly, in our analysis a significant difference in fraction of the rich-club resected was only found for functional networks derived from epochs containing seizures. This is in line with our previous analysis in which the effect of resections could be predicted based on functional networks derived from seizure epochs [42]. Furthermore, our previous study utilised functional networks derived from a nonlinear channel association measure (the surrogate corrected mutual information [62,63]), and we have therefore demonstrated that information relevant to the ictogenic network can be extracted from spatiotemporal seizure dynamics using both linear and nonlinear measures to infer the connectivity structure between nodes. Previous analyses of scalp EEG or MEG have demonstrated that information relevant to epilepsy is also present in background epochs (i.e. those not containing seizures) [22,23,35,38,59,72]. In particular, the predictive power of models for resective surgery has also been studied using inter-ictal (e.g. away from seizure) epochs [43]. Therefore future work should seek to ascertain whether information capable of guiding surgical strategies can be extracted from background data recorded using iEEG. Additionally, it is necessary to examine what neuroimaging modalities contain the most significant information to infer the ictogenic network.

For our theoretical analysis of the impact of network structure on ictogenicity we quantitatively compared both the canonical model and the full neural mass (Wendling model) [37,46]. Our analysis demonstrated that the canonical model, which is essentially a normal form representation of the SNIC bifurcation present in the neural mass model, is a useful parsimonious model, particularly for the purpose of finding the distribution of node ictogenicity (*NI*). In fact, we found that the distribution of *NI* across nodes of a network is almost independent from the particular choice of parameters for sufficiently small networks (*N* < 30). For larger networks (*N* = 50) whose topology yields a non-uniform distribution of *NI*, the two models also return similar predictions of *NI* without the need for parameter calibration. This implies that *NI* depends predominantly on the presence of a bifurcation to spiking dynamics and network structure. We have further shown that the computational gain increases nonlinearly with increasing network size and it is therefore significant for networks such as those inferred from iEEG. The reduction of complexity of models to study fundamental mechanisms of epilepsy [9,74,75] or healthy brain dynamics [76,77] is becoming a well-accepted approach, and will be particularly important in computationally intensive applications, such as the study of perturbations to high-dimensional networks.

Although here we have focused on noise-driven models close to a SNIC bifurcation to generate relevant dynamics, previous studies have suggested the use of other bifurcations, such as saddle-node and homoclinic bifurcations to model seizure onset and offset, respectively [75]. In particular, Jirsa *et al*. [75] used a data-driven approach to identify these bifurcations, under the assumption of a slowly changing control variable moving the model through parameter space. Further work is required to understand to what extent can data reveal which bifurcation underlies the observed dynamical transitions and how different bifurcation mechanisms can influence on the quantification of *NI*. Patient-specific assessment of the type of bifurcation that best describes the data may lead to further improvements of this modeling framework.

We demonstrated that networks with high degree variance are more likely to seize for relatively smaller coupling strengths, whereas more homogeneous networks reach *BNI* = 1 within a more confined range of coupling strengths. This provides a potential explanation for observations of increased degree variance in functional networks derived from epilepsy patients, compared to healthy controls [23]. We also uncovered differences in the interplay between global and local spiking generating mechanisms in networks with different topologies: random and small-world networks display a switch-like mechanism for the emergence of spiking dynamics with respect to changes in global coupling, but a more gradual response to removal of nodes. Nodes in random or small-world networks have smaller degree variance, whereas nodes in scale-free or rich-club networks are more heterogeneous in their degree. Therefore, ictogenicity in the latter networks is likely to be concentrated within a few nodes, and thus larger connectivity strengths are required for spiking dynamics to be present in the whole network, which is required here for *BNI* to be large. These results are in agreement with findings that the critical coupling for the Kuramoto model decreases as the exponent of scale-free networks decreases [78,79]. It is important to note that we assumed all nodes equivalently excitable to focus on the contribution of the network structure to the emergence of spiking dynamics. Future work should consider the potential existence of pathological nodes with higher excitability that may drive the ictogenicity of the network and whose resection may be preferable.

Our analysis suggests that if a brain network under consideration does not have rich-club organization, or if the rich-club were to overlap with eloquent cortex, a resection of a much greater number of nodes would be required. Note that in this context the brain network mapped from iEEG does not correspond to the whole brain, instead it corresponds to a clinically predetermined brain region under investigation as potential surgical targets. Interestingly, our results suggest that a considerable *BNI* reduction could be attained in most 64 node networks upon removal of 10 nodes at random in all topologies, which is comparable to the average epilepsy resection [62]. This is in agreement with findings that most patients undergoing surgery experience some reduction in seizures, even if they do not achieve seizure freedom [80]. In some cases, the rich-club may comprise non-adjacent nodes making it difficult to resect it through surgery. To tackle these cases, other techniques might be considered such as radiofrequency thermocoagulation [81]. Our approach may be improved by quantitatively assessing predictions for changes in seizures frequency, based on the baseline seizures rates of the individuals.

Interestingly, our findings regarding undirected networks did not extend readily to directed networks. In particular, graph theoretical properties of nodes in directed networks did not correlate with *NI*, and the effect of node removals was found to be smaller than it would have in “similar” undirected networks. This has important implications for the choice of network representation of the brain used in studies of perturbations. Depending on the data modality under consideration, different approaches should be considered: for modalities that give rise to undirected networks our framework suggests to target nodes according to their eigenvector centrality, whereas if directed networks are derived it is necessary to assess *NI* using a model (such as the CM). Ultimately, a representation of the brain will be deemed (clinically) useful in the context of our study if it is able to predict the outcome of perturbations. In the current study and our previous work [42] we have demonstrated that useful information is present in an undirected network representation of the brain. However, future work will ascertain whether approaches yielding a directed network may ultimately prove most beneficial.

## Supporting information

S1 Fig *NI* of different directed network topologies in the CM. Each row corresponds to a different network topology: (a)-(c) random network; (d)-(f) scale-free network (SF) (*γ* = 3); (g)-(i) small-world network (SW); (j)-(l) rich-club (RC) network. In the first column the nodes are sorted by their *NI*, so that *NI* is monotonically increasing; in the second column *NI* is sorted by the product of in- and out-degree; and in the third column *NI* is sorted by the dynamical importance (DI) of the nodes. Note that the correlation between these node measures and *NI* is not as good as the one found in Fig 5 for undirected networks. The shaded areas in the first column and the dots in the other panels correspond to 10 different network realizations of the same topology (the line represent the mean). Parameter choices are as in Fig 4.
(TIF)
