# Supplementary figures and images for: An optimal strategy for epilepsy surgery: Disruption of the rich-club?
Source: PLoS Comput Biol. 2017 Aug 17;13(8):e1005637. doi: 10.1371/journal.pcbi.1005637 (PMC5560820; doi:10.1371/journal.pcbi.1005637)

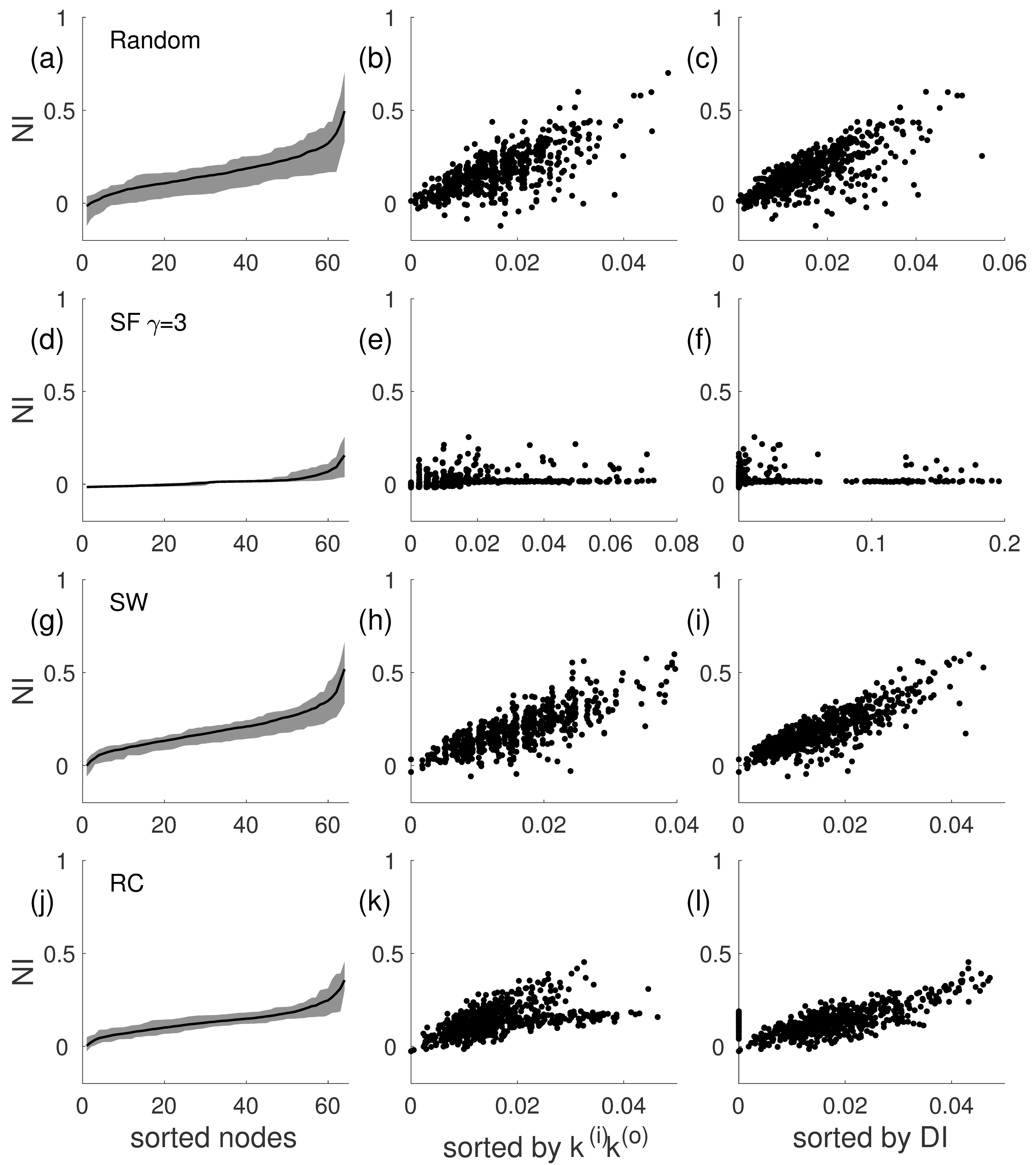

Supplement: S1 Fig — Each row corresponds to a different network topology: (a)-(c) random network; (d)-(f) scale-free network (SF) (γ = 3); (g)-(i) small-world network (SW); (j)-(l) rich-club (RC) network. In the first column the nodes are sorted by their NI, so that NI is monotonically increasing; in the second column NI is sorted by the product of in- and out-degree; and in the third column NI is sorted by the dynamical importance (DI) of the nodes. Note that the correlation between these node measures and NI is not as good as the one found in Fig 5 for undirected networks. The shaded areas in the first column and the dots in the other panels correspond to 10 different network realizations of the same topology (the line represent the mean). Parameter choices are as in Fig 4. (TIF) [file pcbi.1005637.s001.tif]
